# *In Vivo* Observation of Structural Changes in Neocortical Catecholaminergic Projections in Response to Drugs of Abuse

**DOI:** 10.1523/ENEURO.0071-17.2018

**Published:** 2018-02-06

**Authors:** Mai M. Morimoto, Shinji Tanaka, Shunsuke Mizutani, Shinji Urata, Kazuto Kobayashi, Shigeo Okabe

**Affiliations:** 1 Department of Cellular Neurobiology Graduate School of Medicine, University of Tokyo, Bunkyo-ku, Tokyo, 113-0033 Japan; 2 Department of Experimental Psychology, University College London, 26 Bedford Way, WC1H 0AP, London, UK; 3 Department of Molecular Genetics, Institute of Biomedical Sciences, Fukushima Medical University School of Medicine, 960-1295 Japan

**Keywords:** axon, catecholamine, *in vivo* imaging, methamphetamine, neocortex, transgenic mouse

## Abstract

Catecholaminergic (dopamine and norepinephrine) projections to the cortex play an important role in cognitive functions and dysfunctions including learning, addiction, and mental disorders. While dynamics of glutamatergic synapses have been well studied in such contexts, little is known regarding catecholaminergic projections, owing to lack of robust methods. Here we report a system to monitor catecholaminergic projections *in vivo* over the timeframes that such events occur. Green fluorescent protein (GFP) expression driven by tyrosine hydroxylase promoter in a transgenic mouse line enabled us to perform two-photon imaging of cortical catecholaminergic projections through a cranial window. Repetitive imaging of the same axons over 24 h revealed the highly dynamic nature of catecholaminergic boutons. Surprisingly, administration of single high dose methamphetamine (MAP) induced a transient increase in bouton volumes. This new method opens avenues for longitudinal *in vivo* evaluation of structural changes at single release sites of catecholamines in association with physiology and pathology of cortical functions.

## Visual Abstract

**Figure:**
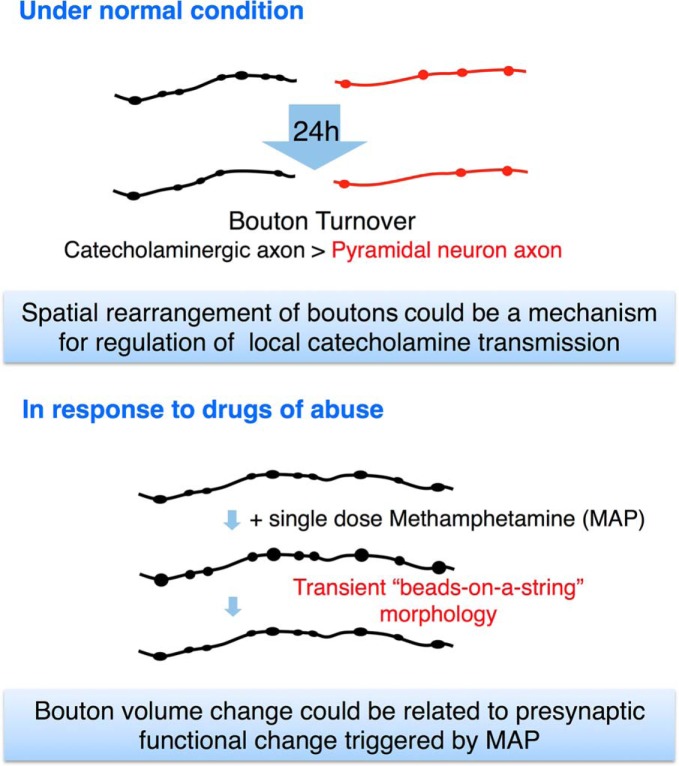

## Significance Statement

This work demonstrates a novel approach for longitudinal two-photon *in vivo* monitoring of catecholaminergic projections in the neocortex using a tyrosine hydroxylase promoter-green fluorescent protein (GFP) transgenic mouse. Using this method, we revealed the highly dynamic nature of the catecholaminergic projections over 24 h period, and a transient morphologic change of these axons in response to a drug of abuse, methamphetamine (MAP). To our knowledge, this is the first observation of such MAP-induced morphologic change of catecholamine axons *in vivo*. In the future, this new method can be extended to observe structural changes associated with cortical development, learning, and mental disorders, in which catecholamine projections to the cortex are known to play a key role.

## Introduction

Neuromodulators, such as monoamines and cholines, exert their functions through axonal projection systems that distribute widely throughout the mammalian brain. Neuromodulators control global brain states including arousal, motivation, mood, and stress. Among them, functional significance of the catecholaminergic system, consisting of dopaminergic and norepinephrinergic neurons, has been well documented (Robbins and Arnsten, 2009). For example, the disruption of their axonal terminals or receptor blockage in the prefrontal cortex (PFC) of monkeys and rats have resulted in impairment of their spatial working memory (Bubser and Schmidt, 1990; Sawaguchi and Goldman-Rakic, 1991; Li and Mei, 1994; Zahrt et al., 1997). Moreover, when catecholamine receptors were stimulated with agonists, PFC neurons’ firing became more finely tuned to relevant stimuli, suggesting a role for catecholamines in the “gating” of signals (Vijayraghavan et al., 2007). Dopamine has classically been known to provide the reward signal in reinforcement learning in the form of “reward prediction error” (Schultz et al., 1997). More recently, synaptic mechanisms have begun to be elucidated, suggesting that temporally precise dopamine signal is necessary for learning (Yagishita et al., 2014).

Catecholamines possess an inverted U-shaped dose-response curve, having an optimal range of concentration for different tasks (Seamans and Yang, 2004). Drugs of abuse, such as methamphetamine (MAP), cocaine, and 3,4-methylenedioxy-MAP (MDMA), act on catecholaminergic systems and raise their extracellular levels to suboptimal high ranges, leading to detrimental effects on cognitive and behavioral states of the subject. Furthermore, antagonists of dopamine and norepinephrine receptors and transporters have been effective in the treatment for major depression, schizophrenia, and attention deficit hyperactive disorder (ADHD), which strongly suggest the alteration of the catecholamine system in these mental disorders (Gamo and Arnsten, 2011). There have been reports of reduced catecholaminergic innervation in postmortem tissues of human patients diagnosed with schizophrenia (Akil et al., 1999) and in a mouse model of schizophrenia (Sekiguchi et al., 2011).

All of these cognitive functions and dysfunctions progress at the time scale of days to months. Longitudinal monitoring of the microstructures of catecholamine neurons could provide insights into synaptic-level mechanisms underlying the progression of these events. While the longitudinal structural imaging of axons and dendrites of pyramidal neurons and some inhibitory neurons have become feasible with *Thy-1*-green fluorescent protein (GFP) transgenic mice and *in utero* electroporation approach (Feng et al., 2000; Lee et al., 2005; Isshiki et al., 2014), imaging of other cell types in the mammalian neocortex has been limited by lack of appropriate genetic methods. More recently, the labeling of different cell types has become possible with the use of Cre-lines and virus mediated methods (Huang et al., 2014; Mastwal et al., 2014). However, with these methods, achieving a homogeneously high and long-lasting stability of expression necessary for longitudinal *in vivo* imaging is generally still a challenge. Also, with some Cre-lines such as tyrosine hydroxylase promoter (*Th*)-Cre, cell type specificity is not guaranteed, often requiring an additional step of confirming the cell type through retrospective immunohistochemistry (Lammel et al., 2015; Stuber et al., 2015).

Here, we developed a method for longitudinal structural imaging of catecholaminergic axons *in vivo* using the *Th*-GFP transgenic mouse. These transgenic mice show high expression and reliable labeling for typical catecholaminergic cell groups [ex. ventral tegmental area (VTA) = 85.2%, substantia nigra pars compacta (SNc) = 94.1%; Matsushita et al., 2002]. We examined the morphology of catecholaminergic axons and their structural changes over 24 h through a cranial window with two-photon microscopy. Quantitation of bouton turnover revealed a higher dynamic fraction for catecholamine neurons compared to Layer II/III pyramidal neurons. During an acute imaging session, a single high dose of MAP induced a transient “beads-on-a-string” morphology of catecholaminergic axons. The present study reveals the details of the catecholaminergic axon dynamics *in vivo* in timescales of minutes to a day in the adult mouse neocortex.

## Materials and Methods

### Animals

For *in vivo* imaging of catecholaminergic axons, transgenic mice expressing GFP under the tyrosine hydroxylase promoter (*Th*-GFP mice) were used (21–31 line; Matsushita et al., 2002). The transgenic line was maintained by crossing to wild type C57BL/6 J inbred mice and heterozygotes aged two to four months were used for the experiments. This *Th*-GFP line labels TH positive neurons in the olfactory bulb, hypothalamus, midbrain and hindbrain regions. Since TH positive neurons in the olfactory bulb and hypothalamus are local neurons and do not project to the cortex (Nagayama et al., 2014; Zhang and van den Pol, 2015), the axons in this study originated mainly from the midbrain (VTA, SNr) and hindbrain (locus coeruleus; LC) regions, which harbor dopamine and norepinephrine neurons projecting to the cortex.

For *in vivo* imaging of pyramidal neuron axons, transgenic mice expressing yellow fluorescent protein (YFP) under the Thy-1 promoter (*Thy-1*-GFP mice, H-line) of similar ages were used (Feng et al., 2000). Both male and female animals were used. All animal procedures were performed in accordance with the University of Tokyo animal care committee’s regulations.

### Surgery

Mice were deeply anesthetized intraperitoneally with ketamine (100 mg/kg) and xylazine (10 mg/kg) diluted in saline. After disappearance of the foot pinch response, the hair of the scalp was shaved, and the scalp over the imaging area was removed. Periosteum tissue was removed with a surgical blade and the frontal or somatosensory area (+2.7 mm from bregma and 0.5 mm lateral from midline, -1.5 mm from bregma and 2.0 mm lateral from midline, respectively) was marked according to stereotactic coordinates. A small metal tube was glued on the skull with dental cement and mice were fixed to the immobilized stage (Narishige) with a heating pad to maintain its body temperature. The skull overlying the frontal cortex (AFC) or somatosensory cortex (SSC) was drilled with a high-speed drill (KM11, Minimo) with a ring type drill bit and carefully removed (Ø 1.8 mm). The dura was covered with a round cover glass (No. 1, Ø 3 mm) and sealed in place with dental cement. After the surgery, *in vivo* time lapse imaging was performed with a two-photon microscope for 3-4 h within the same day, or 1-2 h in consecutive 2 days.

### *In vivo* two-photon imaging

A scanning microscope (FV-300, Olympus) equipped with a mode-locked Ti: Sapphire laser (MaiTai HP, Spectra Physics) was used for imaging with a water immersion objective lens (1.05 NA, 25×, Olympus). The wavelength was 920 nm, and the average power of the laser after the objective lens was between 30 and 40 mW. The imaging area was 234 × 234 μm (low magnification) or 78 × 78 μm (high magnification), with an imaging depth 100 μm from the surface of the neocortex (Layer I–II), and the step size of the z-stack set to 1.0 μm. The image sizes of single horizontal images were set to 512 × 512. For repetitive imaging between days, low magnification images and images of the surface vasculature pattern were taken with a CCD camera (GZ-MG70, Victor), which served as the reference maps for the acquisition of higher magnification images from the same cortical area. The mice were kept on the heating pad until they recovered from the anesthesia and were returned to their home cage.

### Drug administration

MAP hydrochloride (Philopon, Dainippon Sumitomo Pharma; catalog number 871151) was diluted with saline at a concentration of 1 mg/ml for 10 mg/kg injection and 0.25 mg/ml for 2.5 mg/kg injection. Mice were subcutaneously injected with a single dose of 2.5 or 10 mg/kg MAP during the imaging session.

### *In utero* electroporation

For visualization of cortical excitatory axonal processes *in vivo*, Layer II/III neural progenitor cells were transfected via *in utero* electroporation. E14.5–E15.5 pregnant mice were deeply anesthetized with pentobarbital. The uterine horns were exposed and ∼1 µl of DNA solution (containing 1.5 µg/µl of β-actin::DsRed2 plasmid) was injected into the lateral ventricle of each embryo via a glass capillary. Subsequently, the head of each embryo was placed between tweezer electrodes (CUY650P5, NEPA GENE), and four-square pulses (duration: 50 ms, frequency: 1 Hz, 29 V) were applied for electroporation (CUY21EDIT, NEPA GENE).

### *In vivo* microdialysis

C57BL/6 J mice were anesthetized and stereotaxically implanted with stainless-steel guide cannula (outer diameter, 0.51 mm; AG-4, Eicom Co) in the frontal cortex (+2.7 mm from bregma and 0.5 mm lateral from midline). After cannula implantation, a stylet was inserted into the guide until the microdialysis experiment, and was left to stabilize for more than a week.

Two hours before microdialysis experiment, the stylet was replaced with a dialysis probe with a 1.0-mm-long semipermeable membrane (outer diameter, 0.31 mm, AI-4-1, Eicom Co). A two-channel fluid swivel device (SSU-20, Eicom Co) was connected to the inlet and outlet of the probe and artificial CSF (147 mM NaCl, 4 mM KCl, 1.2 mM CaCl_2_, and 0.9 mM MgCl_2_) was infused through the probe at a rate of 1.0 μl/min using a microdialysis pump (CMA/102, Carnegie Medicin). Each mouse was maintained individually in its cage and the dialysis was performed under ketamine (100 mg/kg) and xylazine (10 mg/kg) mixture anesthesia. Following equilibrium period (>2 h), 10 samples were collected every 20 min in vials containing an equal volume (20 μl) of acetic acid solution (40 mM) with EDTA (200 μM). The first four samples were baseline samples. Immediately after collection of the last baseline sample (after 80 min), a single dose of MAP (10 mg/kg) was injected subcutaneously. Samples were stored at –70°C until the HPLC assay.

Dopamine concentration in the dialysate was quantified by HPLC with a pump system (EP-300; Eicom Co). Each sample was injected into a separation column (Eicompak CAX, Eicom Co) with a mobile phase consisting of 0.1 M ammonium acetate buffer at pH 6.0, 0.13 mM EDTA, 50 mM sodium sulfate, and 30% methanol. Samples were then separated out with a separation column maintained at 35°C and detected with an electrochemical detector (ECD-300, Eicom Co). The electrode potential was set to 400 mV against an Ag/AgCl reference electrode. The changes in electric current (nA) were recorded using an integrated data processor (Power Chrom EPC-500, Eicom Co). The dopamine concentration in the dialysate was calculated by reference to the peak area of the standard solution.

### Image and data analysis

All image analyses were done using National Institutes of Health ImageJ and Fiji software (http://rsb.info.nih.gov/ij) and custom-written MATLAB scripts (Mathworks).

For the 24 h repetitive imaging data, the same axonal segments (30-80 μm) were identified from the image stacks from their three-dimensional location and the gross morphology of the axon, which did not change greatly over the course of a day. For quantification of bouton turnover, z-stack images containing only the axon segment of interest were sum intensity projected to include the whole arbor. This prevented any superimposing structures to be included in the projected image. We reconstructed the *xz* plane of these stacks to check the presence of potential artifacts due to movements. Such artifacts would appear as discontinuities in the *xz* plane. We discarded datasets with such artifacts. Next, background intensity was subtracted from the whole image and the axon of interest was traced manually with a line selection tool in ImageJ. The intensity along the selected line was measured. Boutons that exhibited a peak intensity >1.5 times the average axon shaft intensity on day 0 were selected for analysis. If this bouton maintained its peak intensity >1.5 times than the axon shaft (baseline), then this bouton was scored as *stable*. If it fell below 1.2 times the baseline, it was scored as a *loss*. New peaks of intensity >1.5 times than baseline on day 1 were scored as *gain* only when it had an intensity below 1.2 times the baseline on day 0. Turnover rates (TORs), the fraction of gained and lost boutons, were calculated as TOR (t_1_, t_2_) = (N_gained_ + N_lost_)/(N(t_1_) + (N(t_2_)), and survival fraction (SF) was calculated from [1-SF (t_2_ – t_1_)]≈TOR (t_1_, t_2_) (Holtmaat et al., 2005). Statistical significance was evaluated using a two-tailed unpaired Student’s *t* test. Differences were considered to be significant for *p* < 0.05.

**Table 1.** Statistical table

	Data structure	Type of test	Power
a	Normal distribution	Two-tailed unpaired Student’s *t* test	*p* = 0.37
b	Normal distribution	Two-tailed unpaired Student’s *t* test	*p* = 1.9 × 10^−8^
c	Normal distribution	Two-tailed unpaired Student’s *t* test	*p* = 9.8 × 10^−7^

Quantification of bouton volume changes by MAP treatment was done using a semiautomated method. First, 3D image of each time point was maximum intensity projected to 2D. This image was made for all time points and assembled into a stack, and registered using the “stackreg” function in ImageJ. Second, the “find maxima” function in ImageJ was used to obtain local maxima point coordinates for automatic bouton detection. Any points that did not overlap with boutons were excluded on visual inspection. Third, this list of local maxima point coordinates was imported into MATLAB, and a 3 × 3-pixel ROI was made around each point on each image in the stack. The ROI mean pixel intensity was calculated to assess the bouton volume change over time. Finally, all data were normalized to the time of MAP administration. Histograms were generated in MATLAB, in which data were pooled from “before,” “after,” and “recovery” time epochs (before = 20–40 min before MAP administration, after = 15–30 min after injection, and recovery = 60–100 min after injection).

## Results

### Morphologic characteristics of catecholaminergic axons in the adult mouse neocortex imaged *in vivo* under normal conditions

**Figure 1.**
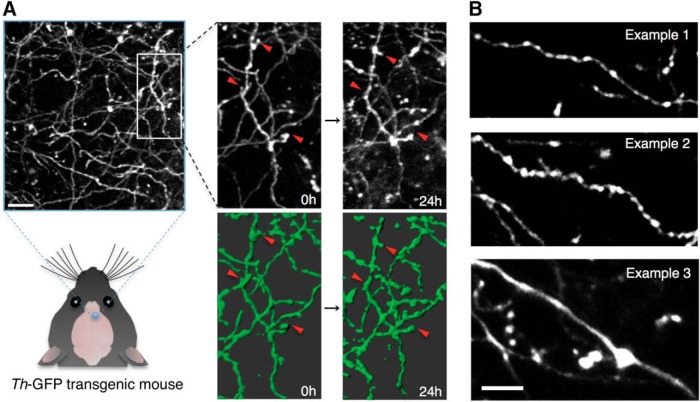
*In vivo* imaging of catecholaminergic axons labeled by *Th*-GFP transgenic line in the mouse neocortex. ***A***, *In vivo* two-photon image of catecholaminergic axons observed in *Th*-GFP transgenic mice. The same axons were repeatedly observed in following experiments. Grayscale images show Z-projections of image stack. Axons in green are 3D-rendered versions. Red arrowheads indicate change events. Scale bar: 15 μm. ***B***, Representative morphology of the major three examples of catecholaminergic axons observed in *Th*-GFP transgenic mice. Most axons were thin and varicose, except for occasional large smooth fibers in deeper layers (∼100 μm). Scale bar: 10 μm.

For visualizing the catecholaminergic neurons *in vivo*, we chose two-photon imaging using a cranial window preparation, which has been shown to be useful for structural imaging in the superficial layers of the cortex (Lee et al., 2005; Holtmaat et al., 2009; Xu et al., 2009). We imaged catecholaminergic axons innervating the Layer I–II (0–100 μm from pia) of the neocortex by two-photon microscope *in vivo* through a cranial window in adult *Th-*GFP transgenic mice. High expression levels of GFP and the relatively sparse innervation of catecholaminergic fibers in the neocortex enabled the clear identification of single axons and their detailed morphology (Fig. 1*A*).

We selected the prefrontal areas of the cortex (frontal association cortex, motor cortex 2, here designated as AFC; Van De Werd et al., 2010) for imaging based on a body of literature indicating catecholamine’s functionally significant role in these areas (Arnsten, 1998; Robbins and Arnsten, 2009). We also imaged in the SSC, where the dynamics of glutamatergic boutons has been reported (De Paola et al., 2006; Holtmaat et al., 2009).

The catecholaminergic axon morphology *in vivo* was not homogeneous. Three notable morphologies were consistently observed (Fig. 1*B*): thin axonal shafts with relatively small boutons and intermediate bouton density (Fig. 1*B*, example 1), thicker axonal shafts with large boutons and high bouton density (Fig. 1*B*, example 2), with occasional side protrusions, and large diameter axonal shafts with very low bouton density (Fig. 1*B*, example 3). Axons similar to examples 1 and 2 were observed in superficial layers (∼50 µm), whereas axons similar to example 3 was only seen in deeper layers (∼100 µm). We observed that while axons similar to example 1 were found in all cortical areas studied (AFC and SSC), axons similar to example 2 were more frequently observed in SSC as compared to the AFC. Axons similar to example 3 were more prominent in the AFC than in the SSC.

These three morphologies closely resembled catecholamine immunoreactive fibers found in fixed brain slices of rats and human cerebral cortex (Berger et al., 1976; Lindvall et al., 1978; Smiley et al., 1992), suggesting a common axonal structure for catecholaminergic cells among the mammalian species.

### One-day interval two-photon time-lapse imaging reveals dynamic structural changes of catecholaminergic axonal boutons and protrusions

**Figure 2.**
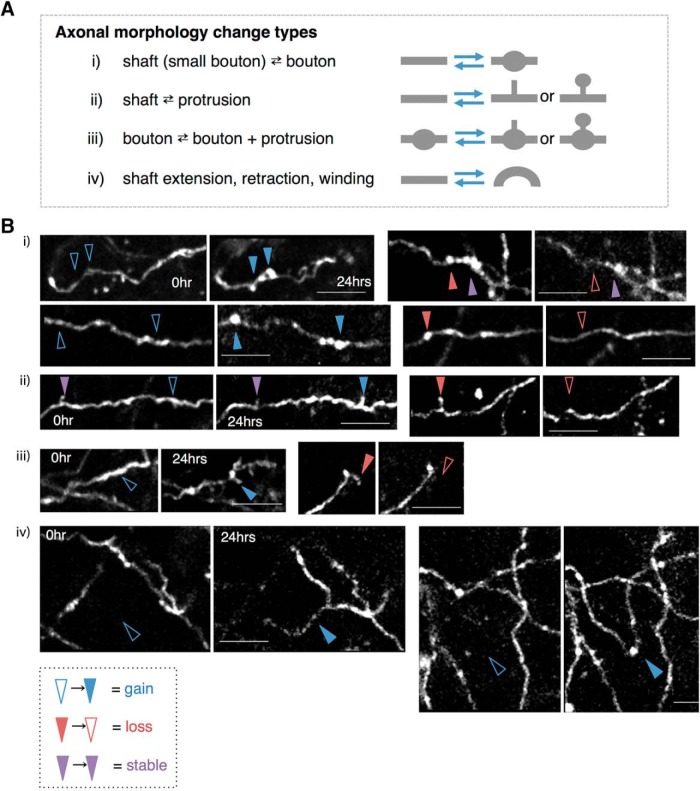
Morphologic changes of catecholaminergic axons over 24 h. ***A***, Types of morphologic changes observed between the two time points (0 and 24 h). ***B***, Examples of the grouped changes i–iv. Blue, red, and purple arrowheads indicate examples of gained, lost, and stable components, respectively.

To explore what structural changes occur in these catecholaminergic axons under unperturbed conditions over the timescale of a day, we obtained stack images of the same area of cortex (the AFC or SSC) each day and compared the morphologies of the axons. A variety of structural changes were observed that could be grouped into four categories (Fig. 2*A*,i–iv). When structural transitions were grouped into these categories (Fig. 2*B*), the loss and gain (turnover) of boutons was the most prominent among them, amounting to ∼77% of all observed changes (Fig. 2*A*). While turnover of boutons and small protrusions have also been previously observed in intracortical projections from pyramidal neurons (De Paola et al., 2006), the very occasional observation of gain or loss of a segment of axonal shaft Fig. 2*A*(iv), *B*(iv)) is a rather surprising event for a 24 h time interval.

**Figure 3.**
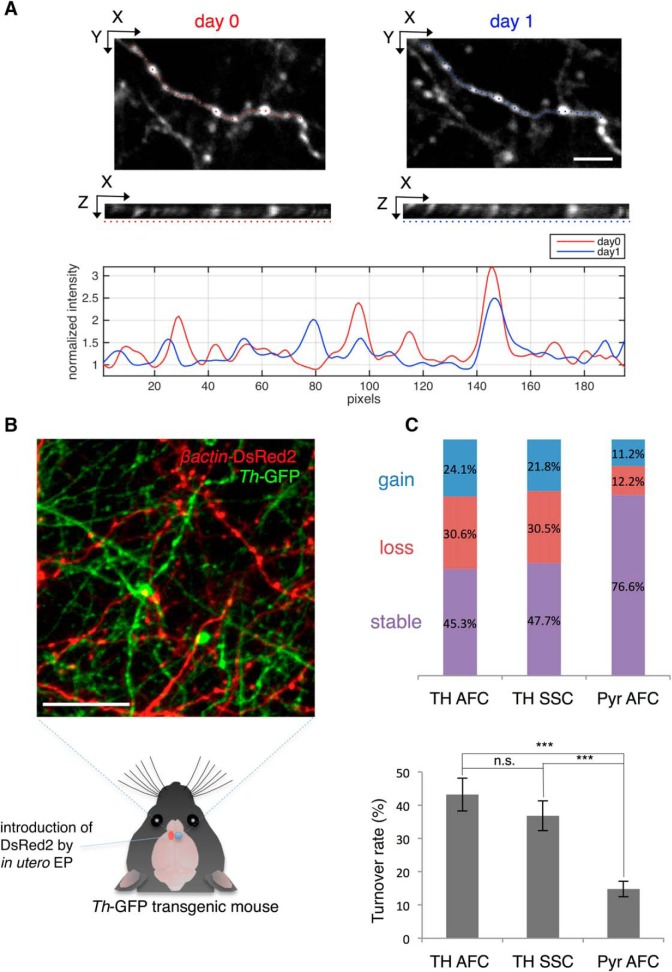
Bouton dynamics of catecholaminergic axons and pyramidal neurons over 24 h. ***A***, Quantification of bouton dynamics over 24 h. Well-isolated axonal arbors were selected for analysis (top: XY plane, middle: XZ plane along the axon; dotted line). Gained, lost, and stable boutons were scored based on the pixel intensity profile along the axon (bottom). Scale bar: 5 μm. ***B***, Observation of Layer II/III pyramidal neuron axons (red) and catecholaminergic axons (green) within the same preparation *in vivo*. Scale bar: 25 μm. ***C***, Top, Fraction of gained, lost, and stable boutons of dopaminergic axons in the AFC (TH AFC), catecholaminergic axons in the SSC (TH SSC), and pyramidal neuron axons in the AFC (Pyr AFC). TH AFC: *N* = 5, *n* = 232; TH SSC: *N* = 5, *n* = 197; Pyr AFC: *N* = 5, *n* = 278. Bottom, Comparison of TORs between axon types. *N*: number of animals, *n*: number of boutons. Data are presented as mean ± SEM; ***p < 0.001, n.s.: not significant.

To quantitate the dynamics of the boutons, we next measured the gain and loss of these boutons. To this end, we randomly selected well-isolated axonal segments from the stack images and compared the positions and numbers of boutons observed at each of the two time-points. Previous reports of turnover of excitatory cell boutons (De Paola et al., 2006; Holtmaat et al., 2009) led us to expect relatively modest changes. In contrast, the dynamic fraction of catecholaminergic boutons was considerably higher (Fig. 3*C*). No significant difference was observed between the two areas studied (AFC and SSC; Fig. 3*C*, Table 1; *p* = 0.37^a^). We controlled for potential confounding effects of the craniotomy on the turnover of boutons by directly comparing the turnover of catecholaminergic axonal boutons with those of glutamatergic axons from pyramidal neurons. By labeling a subset of pyramidal neurons with a red fluorescent protein (DsRed2 driven by β-actin promotor) by *in utero* electroporation, both types of axons could be compared within the same transgenic mouse preparation (Fig. 3*B*). Quantitation of bouton turnover revealed a significantly larger dynamic fraction of boutons for catecholaminergic axons compared to pyramidal cell axons in the PFC (Fig. 3*C*, Table 1; TH AFC: *p* = 1.9 × 10^−8b^, TH SSC: *p* = 9.8 × 10^−7c^) . The SF of Layer II/III pyramidal neuron boutons in our study over 24 h was 85.3%. Importantly, this value was comparable with a previous report (∼90%; De Paola et al., 2006). These data argued that craniotomy did not significantly impact the structural dynamics of catecholaminergic axons, and that the structural dynamics of presynaptic boutons is differentially regulated between glutamatergic and catecholaminergic axons within the same cortical area.

### High-dose MAP administration induces a transient beads-on-a-string morphology of catecholaminergic axons

Drugs of abuse, such as MAP, cocaine or MDMA, act on monoaminergic systems and increase the extracellular level of monoamines in the neocortex (Darvesh et al., 2011). These drugs also promote structural changes in the density of dendritic spines after prolonged administration in the rat neocortex (Robinson and Kolb, 2004; Koda et al., 2010). Would these drugs that alter the release of catecholamines affect the structure of catecholamine axons *in vivo*?

**Figure 4.**
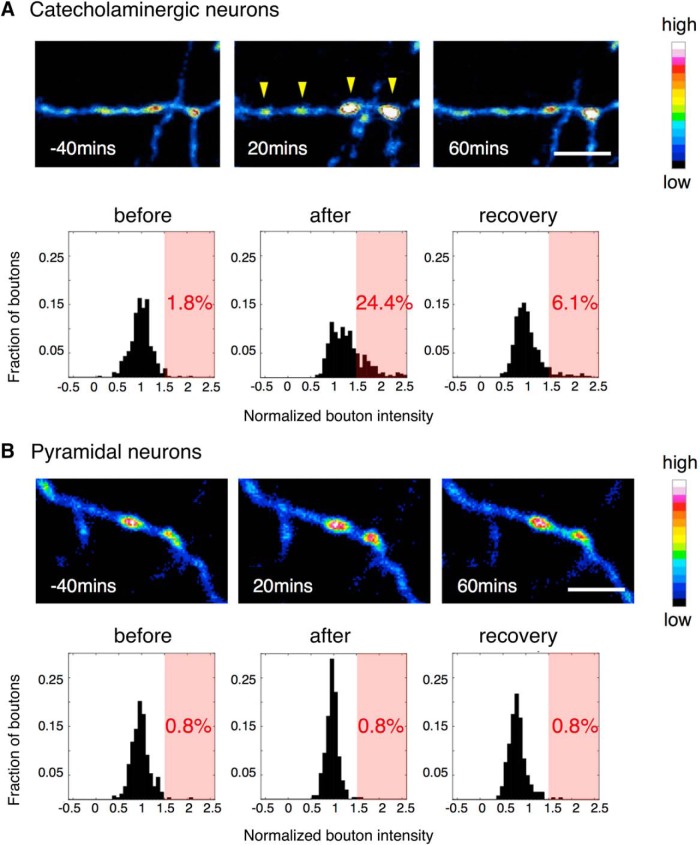
Morphologic change of catecholaminergic boutons after MAP injection in the AFC. A single dose of MAP (10 mg/kg) induced a transient beads-on-a-string morphology in catecholaminergic neurons (***A***), while in pyramidal neurons, there was no obvious structural change (***B***); yellow arrowheads: enlarged boutons; scale bar: 10 μm. The histograms show bouton intensity normalized to MAP injection time point. After injection, the distribution of catecholaminergic bouton intensity shifted to above 1.5 times its original intensity (light red background), while pyramidal neuron boutons did not. Catecholamine neurons: *N* = 5, *n* = 393; pyramidal neurons: *N* = 5, *n* = 263 (*N*: number of animals, *n*: number of boutons).

A single dose of MAP (2.5 or 10 mg/kg) was administered subcutaneously during the imaging session. Stack images were obtained in intervals of 5-20 min. While a low dose administration (2.5 mg/kg; data not shown) did not visibly change axonal structures, a high dose administration (10 mg/kg) induced a transient, but pronounced beads-on-a-string morphology in axons (Fig. 4*A*). This morphologic transition was characterized by an enlargement of the bouton diameter. We quantified the pixel intensity of the bouton as an index of its volume and analyzed the distribution of the intensities (normalized to the time of injection) in time epochs before injection of MAP, after injection, and after a recovery period. The catecholamine bouton intensities exhibited a skewed trend toward higher intensity after the injection (Fig. 4*A*). The fraction of boutons that increased in volume over 1.5 times after MAP injection reached 24.4%, indicating that a significant portion of boutons increased in volume. However, this distribution returned to near baseline levels after the recovery period.

To investigate whether this bouton volume increase induced by MAP is specific to catecholamine neurons, we conducted the same experiment on H-line mice, which express YFP in a subset of pyramidal neurons. In contrast to our previous results, these mice did not exhibit a clear change in the distribution of the bouton intensities after injection of MAP (Fig. 4*B*). Instead the pyramidal neurons seemed to show a different effect in which the volume change of the boutons were generally decreased after MAP injection, resulting in a narrower distribution around 1. We also controlled for the potential catecholaminergic neuron specific effects of the used anesthetics (ketamine and xylazine) by conducting the same experiment on an animal under a different anesthetic (isoflurene). We obtained similar results, and therefore did not find evidence for confounding effects of ketamine and xylazine (data not shown).

**Figure 5.**
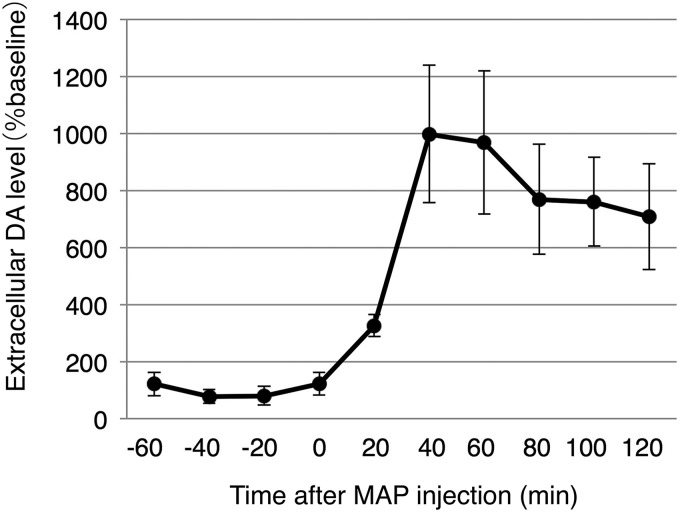
Extracellular dopamine concentration measured by microdialysis in the AFC. Mice were kept under anesthesia in an identical condition to the *in vivo* imaging experiments. A single dose of MAP (10 mg/kg) was injected at time 0 and was monitored in 20 min intervals. *N* = 4 mice.

These results suggested that the bouton volume increase induced by MAP are likely mediated through molecular processes specific to catecholaminergic neurons. For example, MAP is known to act on DATs (dopamine reuptake transporters) expressed in dopamine neurons, to reverse its transport direction resulting in dopamine release (Sulzer et al., 2005). Indeed, after MAP injection, extracellular dopamine concentration (as measured by *in vivo* microdialysis during the same MAP injection condition) peaks within 1 h, and decays thereafter (Fig. 5). The timing of the bouton volume increase precedes this observed rise of the dopamine concentration. These results support a model in which MAP triggers the transient bouton volume increase in the catecholamine neurons leading to the increase in the extracellular levels of dopamine. While the functional significance of this morphologic change is currently unknown, the changes may contribute to processes that underlie cognitive impairments, such as hallucination and psychosis experienced by high dose drug abusers (Wood et al., 2014).

## Discussion

In this study, we observed the *in vivo* structural dynamics of catecholaminergic innervation to the neocortex, which are known to play a significant role in distinct cognitive functions as well as mental disorders.

The following were major findings:

1) Time lapse imaging over 24 h under normal condition revealed various morphological changes in catecholamine axons ranging from loss and gain of boutons and protrusions, to changes in the winding of the whole arbor (Figs. 1*A*, 2). The TORs of AFC and SSC boutons were both significantly higher than that of AFC Layer II/III pyramidal cell boutons. However, no significant difference was seen for catecholaminergic boutons between regions, suggesting an overall dynamic nature of catecholamine release sites in the neocortex (Fig. 3).
2) When a single high dose MAP was injected during the imaging session, we observed a transient beads-on-a-string morphology of the axon (Fig. 4), which preceded the rise of extracellular DA concentration under the same condition (Fig. 5). This structural modification was found in catecholamine axons, but not in Layer II/III pyramidal cell axons (Fig. 4), suggesting a cell type-specific mechanism.

### High TOR of catecholaminergic axonal boutons

Our analysis of bouton turnover yielded a higher TOR (∼50% dynamic fraction) for catecholaminergic axonal boutons compared to pyramidal cell axonal boutons (∼20% dynamic fraction; Fig. 3*C*). Such TOR of catecholaminergic axonal boutons seems constant even over a longer period (preliminary data; *N* = 3 mice, 41 out of 80 boutons persisting over 4 d). This suggests the existence of two distinct pools of boutons that have longer and shorter lifetimes, respectively.

The higher turnover of the observed catecholaminergic boutons could be due to the fact that most monoaminergic presynaptic release sites do not make synapses in the neocortex (Descarries et al., 1975), and uses the so-called “volume transmission” as their main strategy of transmission (Fuxe et al., 2010). Therefore, it can be hypothesized that, as a consequence of the presynaptic sites not being adhered to the postsynapse, the presynapse is not able to maintain itself at a specific location on the axonal shaft, and thus has a larger dynamic fraction. Another possibility could be that catecholamine boutons are induced or eliminated at certain locations in response to some molecular cue, to shift the site of local catecholaminergic transmission depending on the time of day or arousal level (Hobson and Pace-Schott, 2002). Whether this high dynamic fraction is due to such passive or active mechanism remains as a subject for future studies.

### Transient beads-on-a-string morphology induced by high-dose MAP administration

Most studies that found structural changes in axonal boutons were conducted *in vitro* and report neurotoxicity of the MAP leading to blebbing of axons and subsequent cell death (Cubells et al., 1994; Larsen et al., 2002). The phenomenon we observed is unlikely to be part of such cell death processes, because the axons returned to its original morphology after a recovery period (Fig. 4).

Morphologic changes of axonal compartments are known to relate to functional changes such as the regulation of transmitter release. Here we observed a significant bouton volume change with a MAP injection, which was specific to catecholaminergic axons. This indicates that the molecular pathways in which MAP affect catecholaminergic neurons result in a bouton volume change as well as the increase in transmitter release (Fig. 5). We therefore speculate that this novel bouton volume change reported here, is part of the mechanism for the increased transmitter release.

We also observed variability in the extent of bouton volume change (Fig. 4), which may reflect some kind of variability in the susceptibility to MAP among boutons. One study which looked at the ultrastructure of striatal dopaminergic boutons in rats treated with MAP daily for two weeks found that the decrease in bouton number was specific to the population of boutons without mitochondria, and the boutons with mitochondria stayed intact (Ihara et al., 1986). Ultrastructural analysis of affected and non-affected boutons may provide mechanistic insights into this structural change in the future.

### Applicability of *Th*-GFP transgenic mice for longitudinal *in vivo* imaging

By exploiting this method for longitudinal *in vivo* imaging, the utility of *Th-*GFP mice can be significantly expanded for a variety of experiments in the future. Catecholamine axons are known to continuously develop for an extended period of time after birth (Pascucci et al., 2007; Segovia et al., 2008). Therefore, imaging of axonal process during early development would provide insights into how catecholaminergic axons integrate into preexisting, or developing cortical circuits. Since learning related structural plasticity is known to occur at dendritic spines (Matsuzaki et al., 2004; Xu et al., 2009), it would be of interest to observe whether the catecholaminergic boutons would also structurally change *in vivo* especially under reinforcement learning paradigms. TH positive projections from the LC to the hippocampus were recently found to mediate novelty-associated memory enhancement (Takeuchi et al., 2016). Therefore, it would also be intriguing to observe the dynamics of the TH projections *in vivo* during the process of such plasticity in the hippocampus using this method. On the other hand, deficits in catecholaminergic innervation are implicated in some mental disorders including schizophrenia (Akil et al., 1999; Sekiguchi et al., 2011). *Th*-GFP mice could be crossed to mouse models of mental disorders to investigate alterations in the structural dynamics of those strains and to correlate differences in the axonal dynamics with behavioral and physiologic changes. Taken together, this new capability to follow dynamic changes of catecholaminergic axons longitudinally would be useful for asking important questions related to the development of cortical circuits, learning related plasticity, and for understanding mechanisms underlying alterations in the catecholamine system that result in mental disorders.

## Synthesis

Reviewing Editor: Pablo Castillo, Albert Einstein College of Medicine Decisions are customarily a result of the Reviewing Editor and the peer reviewers coming together and discussing their recommendations until a consensus is reached. When revisions are invited, a fact-based synthesis statement explaining their decision and outlining what is needed to prepare a revision will be listed below. The following reviewer(s) agreed to reveal their identity: Anthony Holtmaat, Wen-Biao Gan

The reviewers agree in that your revised manuscript has been improved significantly. However, as you will see below, they have also made a few comments that you should fully address before your paper can be published in eNeuro. Reviewer #1 requests additional clarifications about quantification and data analysis. Reviewer #2 requests that you include the 4-day imaging data. You have 30 days to revise your manuscript.

REVIEWER #1

The paper has been improved. The authors now provide more details and give a bit more insights into the dynamics. The number of observations have been increased and the statistics are therefore a bit stronger. The main improvement possibly concerns the bouton intensity measurement replacing the visual inspection-based analysis in the initial manuscript.

There are still a few issues that I find concerning, and that need to be resolved before publication.

1.I appreciate the explanation of the authors regarding the classification of the axons, as well as their reasons for not further analyzing the origin of the differences between axons, but why then provide the percentages in Figure 2A. First of all, it is completely unclear how the authors got to these numbers. Over what length did an axon have to fulfill criteria i, ii, iii, or iv in order to be classified as such? If the authors do not follow up on this information (e.g. compare with other axon types) and do not use it in the subsequent analysis of turnover, why then give these percentages? The classification suffices as currently done in the text. I think that the percentages can be removed from Fig. 2A. It adds more to confusion than that it resolves anything.

2.The more careful analysis based on bouton intensity in Fig. 3 is a clear improvement. I have a hard time understanding though as to why with the new 1.5x baseline criterion for bouton detection (vs the 2.5x in the original submission) the percentage of turnover hardly deviates from the original numbers. According to Fig. 3A the authors should detect many more boutons with the new method - and possibly also see much more turnover in Fig. 3B. Can the authors explain this?

3. The examples that are given for micro-scale changes in Fig. 1A remain somewhat odd. The problem being that the authors zoom in on a few small remodeling terminals, while in the background entire axonal arbors appear or disappear. If this is representative of the whole data set the authors need to explain this in much more detail, and provide support for why they only analyzed bouton size changes in response to MAP, and decided not to analyze branch removal or addition; with the latter possibly being a much more likely source for large fluctuations in DA than bouton size changes

REVIEWER #2

The authors have addressed my previous concerns satisfactorily - particularly with regards to the mouse line, method of quantification, and the limited number of animals. The preliminary data that 17 out of 27 boutons persists after four days is interesting and could not be predicted from the high turnover rate from the one-day imaging experiment. I suggest that the authors include this finding in the manuscript.
